# Multidimensional effects of voice therapy in patients affected by unilateral vocal fold paralysis due to cancer^☆^

**DOI:** 10.1016/j.bjorl.2017.07.012

**Published:** 2017-08-24

**Authors:** Camila Barbosa Barcelos, Paula Angélica Lorenzon Silveira, Renata Lígia Vieira Guedes, Aline Nogueira Gonçalves, Luciana Dall’Agnol Siqueira Slobodticov, Elisabete Carrara-de Angelis

**Affiliations:** a AC Camargo Cancer Center, Fonoaudiologia, São Paulo, SP, Brazil; b AC Camargo Cancer Center, Departamento de Otorrinolaringologia e Cirurgia de Cabeça e Pescoço, São Paulo, SP, Brazil

**Keywords:** Voice disorders, Vocal fold paralysis, Voice Therapy, Therapy, Distúrbios vocais, Paralisia das pregas vocais, Terapia da voz, Reabilitação

## Abstract

**Introduction:**

Patients with unilateral vocal fold paralysis may demonstrate different degrees of voice perturbation depending on the position of the paralyzed vocal fold. Understanding the effectiveness of voice therapy in this population may be an important coefficient to define the therapeutic approach.

**Objective:**

To evaluate the voice therapy effectiveness in the short, medium and long-term in patients with unilateral vocal fold paralysis and determine the risk factors for voice rehabilitation failure.

**Methods:**

Prospective study with 61 patients affected by unilateral vocal fold paralysis enrolled. Each subject had voice therapy with an experienced speech pathologist twice a week. A multidimensional assessment protocol was used pre-treatment and in three different times after voice treatment initiation: short-term (1–3 months), medium-term (4–6 months) and long-term (12 months); it included videoendoscopy, maximum phonation time, GRBASI scale, acoustic voice analysis and the portuguese version of the voice handicap index.

**Results:**

Multiple comparisons for GRBASI scale and VHI revealed statistically significant differences, except between medium and long term (*p* < 0.005). The data suggest that there is vocal improvement over time with stabilization results after 6 months (medium term). From the 28 patients with permanent unilateral vocal fold paralysis, 18 (69.2%) reached complete glottal closure following vocal therapy (*p* = 0.001). The logistic regression method indicated that the Jitter entered the final model as a risk factor for partial improvement. For every unit of increased Jitter, there was an increase of 0.1% (1.001) of the chance for partial improvement, which means an increase on no full improvement chance during rehabilitation.

**Conclusion:**

Vocal rehabilitation improves perceptual and acoustic voice parameters and voice handicap index, besides favor glottal closure in patients with unilateral vocal fold paralysis. The results were also permanent during the period of 1 year. The Jitter value, when elevated, is a risk factor for the voice therapy success.

## Resumo

**Introdução:**

Pacientes com paralisia unilateral de prega vocal podem apresentar diferentes graus de distúrbios da voz, dependendo da posição da prega vocal paralisada. A compreensão da eficácia da terapia vocal nesta população pode ser um coeficiente importante para definir a abordagem terapêutica.

**Objetivo:**

Avaliar a eficácia da terapia vocal em curto, médio e longo prazos em pacientes com paralisia unilateral de prega vocal e determinar os fatores de risco para falha na reabilitação da voz.

**Método:**

Estudo prospectivo, no qual 61 pacientes com paralisia unilateral de prega vocal foram recrutados. Cada participante foi submetido a terapia vocal com um fonoaudiólogo experiente duas vezes por semana. Um protocolo de avaliação multidimensional foi utilizado no pré-tratamento e em três momentos após o início da terapia da voz: curto prazo (1-3 meses), médio prazo (4-6 meses) e longo prazo (12 meses); incluiu videoendoscopia, tempo máximo de fonação, escala GRBASI, análise de voz acústica e a versão em português do *Voice Handicap Index*.

**Resultados:**

Os dados comparativos temporais das avaliações revelaram diferenças estatisticamente significativas, exceto entre médio e longo prazo (*p* < 0,005). Os dados sugerem que há melhora vocal ao longo do tempo com resultados de estabilização após seis meses (médio prazo). Dos 28 pacientes com paralisia unilateral permanente da prega vocal, 18 (69,2%) atingiram o fechamento glótico completo após a terapia vocal (*p* = 0,001). O método de regressão logística indicou que o Jitter entrou no modelo final como um fator de risco para melhora parcial. Para cada unidade de aumento de Jitter, houve um aumento de 0,1% (1,001) da chance de melhora parcial, o que significa um aumento na chance de não ocorrer melhora completa durante a reabilitação.

**Conclusão:**

A reabilitação vocal melhora os parâmetros de voz perceptiva e acústica e o índice de incapacidade vocal, além de favorecer o fechamento glótico em pacientes com paralisia unilateral da prega vocal. Além disso, os resultados também foram permanentes durante o período de um ano. O valor de Jitter, quando elevado, é um fator de risco para sucesso parcial da terapia vocal.

## Introduction

Unilateral vocal fold paralysis (UVFP) may present as dysphonia, loss of the upper register of the voice, hoarseness, breathiness, throat pain, choking episodes or decreased vocal stamina.^1^, ^2^, ^3^, ^4^, ^5^ Patients with UVFP may demonstrate different degrees of voice perturbation depending on the position of the paralyzed vocal fold.^6^, ^7^, ^8^

Treatment of unilateral vocal fold paralysis is designed to eliminate aspiration and improve quality of the voice. Different surgical techniques are available today: teflon, collagen, hydroxiapatite or autogenous micronized dermis, fat injection, type I thyroplasty and nerve muscle pedicle transfer represent the surgical techniques mainly adopted. These studies conduct evaluations mostly in the immediate post-surgery period and three months after surgery.^9^, ^10^, ^11^, ^12^, ^13^, ^14^, ^15^, ^16^, ^17^, ^18^

Only three studies have evaluated voice therapy efficacy for this population. Schindler et al.^19^ analyzed retrospectively voice modifications in 40 patients with UVFP from different etiologies before and after voice therapy. A multidimensional assessment protocol was used and it included videoendoscopy, the maximum phonation time (MPT), the GRBASI scale, spectrograms, perturbation analysis and the voice handicap index (VHI). Pre and post early treatment data were compared. A complete glottal closure was seen in 8 patients before voice therapy and in 14 afterwards. Mean MPT increased significantly. In the perceptual assessment, the difference was significant for five out of six parameters and a significant improvement on quality of life was also reached. D’Alatri et al.^20^ evaluated the laryngological and acoustical results obtained after voice therapy in 8 patients with UVFP caused by different etiologies. After behavioral therapy, the prevalence of complete glottal closure increased significantly (*p* < 0.05). Subjects’ pre-therapy mean values for Jitter, Shimmer and noise-to-harmonic ratio were significantly different from those taken both immediately and 6 months after treatment (*p* < 0.05). Mattioli et al.^21^ conducted a 7 years prospective study to evaluate the post vocal early treatment results of 74 patients with UVFP. Patients underwent multidimensional assessment pre and post treatment and the results shown that 51 (68.9%) patients recovered vocal fold mobility, and 23 (31.1%) had persistent paralysis after voice therapy. In this group of patients, complete glottal closure was observed in 5 cases before the voice therapy, and in 13 patients this complete closure was observed only after the therapeutic process (*p* < 0.0001). An important and significant reduction in fundamental frequency was found (*p* < 0.0001); an improvement was seen for the mean values of Jitter (Jitt%; *p* = 0.001), Shimmer (Shim%; *p* < 0.0001) and noise-to-harmonic ratio (NHR) (*p* < 0.0001). Voice handicap index (VHI) values showed a clear and significant improvement and mean MPT increased significantly.

At this time, there are no studies that report the risk factors for vocal improvement after voice therapy. Although there are three previous studies demonstrating the efficacy of voice therapy for patients with UVFP, there are no multidimensional studies that evaluate the long term.

Understanding the effectiveness of voice therapy in this population may be an important coefficient to define the therapeutic approach. Voice therapy is the noninvasive intervention and an understand of factors listed above is essential for adequate indication. This is the first study that evaluated the voice therapy effectiveness for short, medium and long term in oncological patients with UVFP and that determine the risk factors for voice rehabilitation failure.

## Methods

### Patients

This study was approved by the Institution Ethics Committee (n° 1399/10). A total of 61 patients affected by UVFP were enrolled in a prospective study. Inclusion criteria included adults with UVFP diagnosis without structural lesions or previous dysphonia. The study group comprised 16 (26.2%) men and 45 (73.8%) women, with a mean age of 52.4 (SD = 13.8) years. The UVFP etiology was due to different types of cancer treatment, including thyroid (44.69%), lung (11.5%), esophagus (5.0%) and seven other different locations (14%).

From the 61 patients included, 43 finished the voice rehabilitation treatment. Nine of them declined, and nine patients had worsening their clinical oncologic condition. After voice therapy, UVFP persisted in 28/43 (65.2%) patients. For multidimensional analysis only the 28 patients who remained with UVFP were recorded.

Concerning clinical analysis, the patients were divided in three groups: “overall improvement” – individuals who had all vocal and acoustic appropriate parameters, improvement in quality of life and no complaints; “partial improvement” – individuals without complaints, however with persistence of discrete changes in individual parameters; “failure” – individuals who did not present vocal, as well as acoustic parameters and/or life quality improvement.

### Multidimensional assessment

Patients were assessed pretreatment and at 3 different times after voice treatment: short term (1–3 months), medium term (4–6 months) and long term (12 months).

#### Laringoscopy

Each subject underwent videolaryngoscopy evaluation with a flexible endoscope. Endoscopic examinations were conducted using a Kay9105 endoscope coupled with a Panasonic GP micro camera (model AD22TA) connected to a Sony14 inch monitor and a Philips DVD-R 335HDD. The larynx was evaluated during breathing and sustained phonation of vowels /e/ and /i/ in randomized order for the assessment time point (pre, short, medium or long term). The examiner was blinded to the timing of the evaluation. On the basis of videolaryngoscopic images, each patient was evaluated according to mobility of the vocal folds and UVFP position. For the evaluation analysis, an adapted protocol was used.^22^, ^23^

#### Perceptual voice analysis

Perceptual evaluation was based on the GRBASI scale.^24^, ^25^, ^26^, ^27^ The vocal samples were computer recorded using the Multi-Dimensional Voice Program (MDVP) from the patient's mouth during the production of a sustained /a/. All of the vocal samples were subsequently evaluated by three experienced judges (speech language therapists) with no knowledge about when the voice was recorded (pre, short, medium or long term). Five days after the first evaluation, 35% of the voices were re-tested. Both results were evaluated using a Kappa test, and the most analogous judgment result was chosen for the study.

#### Maximum phonation time

This was obtained during a sustained /a/ at comfortable pitch and loudness after a profound inspiration. Three consecutive trials were performed, and the best one was considered.

#### Acoustic voice analysis

Acoustic voice analysis was performed with a Multi-Dimensional Voice Program (MDVP, Kay Pentax). Subjects were asked to sustain the vowel /a/ at a comfortable pitch and loudness level for at least four seconds. A three-second interval was selected, eliminating the beginning and the final emission. The following parameters were considered: mean fundamental frequency (Mean F0, hertz), Jitter%, PPQ, Shimmer%, APQ, fundamental frequency variation (vFo), amplitude variation (vAm) and Voice Turbulence Index (VTI).

#### Self-assessment

Finally, each patient completed the Brazilian version of the VHI to have self-assessment data on voice related quality of life.

### Voice therapy

Voice therapy was individualized, two times per week, for 30 minutes. Each patient involved in the study had several voice therapy sessions with an experienced speech language therapist. The mean number of sessions was 12 (SD = 6.1). The voice therapy aimed to improve glottal closure and at the same time avoid undesirable compensatory behaviors, such as anterior-posterior or lateral constriction of the vocal folds, falsetto voice and oral or pharyngeal muscle tension.

At the first session, the speech-language pathologist provided patients with educational information about the workings of phonation, their specific abnormality, and vocal hygiene.

The patients were submitted to specific voice training, directed toward avoidance of hyper functional compensation: vocal fold vibration and forced adduction exercises, such as pushing or pulling on chair. Patients who used falsetto register compensation were oriented to bring out the chest voice by moving the larynx to lower position in the neck (manually or using techniques such as deep inhalation and yawning). For patients with ventricular hyper function, we used techniques such as speaking on inhalation and nasal sounds.^8^, ^28^, ^29^, ^30^, ^31^, ^32^, ^33^

All the therapeutic processes were conducted with visual feedback of the acoustic parameters to help choose the best technique for therapy, as well as to facilitate patient results observation.

### Statistical analysis

Statistical analyses were performed using the SPSS 11.5 package (SPSS Science, Chicago, IL). For quantitative variables, the Chi-Square and Fisher's exact test (*F*) were used. For comparison between two groups, Student's *t* test was used. Analysis of variance (ANOVA) was used for other analyses. When homogeneity of variances was not verified, adjustment was performed with the Brown–Forsythe (BF) test.

To evaluate the longitudinal characteristics, the Repeated Measures for Ordinal Data test was used. When differences were identified between the evaluations, multiple comparisons were made specifically for this test.

When there was a significant difference between times, to identify differences in evaluation time points, comparisons were made between the two time points using the Bonferroni test. These comparisons are presented at the end of Table 1, indicating when the *p*-value <0.05 (significance level considered was 5%).

**Table 1:** Comparison of clinical assessment values at the four time points.

Categories	PT	ST	MT	LT	*p*
	*n* (%)/data	*n* (%)/data	*n* (%)/data	*n* (%)/data	
*G*	(*n* = 28)	(*n* = 28)	(*n* = 14)	(*n* = 7)	
Normal	0	8 (28.6)	6 (42.8)	4 (57.1)	
Changed	28 (100)	20 (71.4)	8 (57.2)	3 (42.9)	0.001

*R*					
Normal	2 (7.1)	7 (25)	6 (42.8)	5 (71.5)	
Changed	26 (92.9)	21 (75)	8 (57.2)	2 (28.5)	0.001

*B*					
Normal	3 (10.7)	11 (39.3)	10 (71.4)	5 (75.5)	
Changed	25 (89.3)	17 (60.7)	4 (28.6)	2 (28.5)	0.001

*A*					
Normal	28 (100)	28 (100)	14 (100)	7 (100)	
Changed	0	0	0	0	NA

*S*					
Normal	28 (100)	28 (100)	14 (100)	7 (100)	
Changed	0	0	0	0	NA

*I*					
Normal	9 (32.1)	16 (57.1)	11 (78.6)	4 (57.1)	
Changed	19 (67.9)	12 (42.9)	3 (21.4)	3 (42.9)	0.001

*Degree of dysphonia*					
Normal	0	8 (25.9)	6 (42.9)	4 (57.1)	
Discrete	5 (17.8)	10 (37.0)	7 (50.0)	2 (28.6)	
Moderate	12 (42.9)	9 (33.3)	1 (7.1)	1 (14.3)	
Severe	11 (39.3)	1 (3.7)	0	0	0.001

*MPT /a/*					
Min–max	2–15	4–17	4–15	5–14	
Median	6	12	11	7	
Mean ± SD	7 ± 3.3	11.1 ± 3.3	10.8 ± 3.6	7 ± 6.2	0.001

*Pitch*					
Normal	20 (71.4)	23(82.1)	13 (92.8)	6 (85.7)	
Changed	8 (28.6)	5 (17.9)	1 (7.2)	1 (14.3)	0.001

*Loudness*					
Normal	9 (32.1)	17 (60.7)	12 (85.7)	6 (85.7)	
Changed	19 (67.9)	11 (39.3)	2 (14.3)	1 (14.3)	0.001

L, overall dysphonia grade; R, roughness; B, breathiness; A, asthenia; S, strain; I, instability; min, minimum; max, maximum; SD, standard deviation; PT, pre-therapy; ST, short term; MT, medium term; LT, long term.

We performed logistic regression analysis to identify the risk factors that influenced the partial and not full improvements. The variables that presented a *p* < 0.10 in the univariate analysis were included in the initial model.

The Stepwise forward logistic regression method was used and does not include variables analyzed jointly in the final model that were not significant. Thus, only the significant variables (*p* < 0.05) or those with a significant trend (0.05 < *p* < 0.10) were included in the final model. All other variables were not included and in all tests, a *p*-value of less than 0.05 was considered significant.

## Results

From the 28 patients with permanent UVFP, 18 (69.2%) presented with complete glottal closure following voice therapy (*p* = 0.001).

Perceptive auditory analysis comparisons pre and post therapy (short, medium and long term) revealed a significant difference between the four evaluation time points for most parameters (overall dysphonia degree, roughness, breathiness, instability, maximum phonation time, loudness and pitch) (Table 1). The multiple comparisons for the GRBASI scale revealed significant differences at the different evaluation time points, except between the medium and long term time points (*p* < 0.005). The data suggests that there was a voice improvement over time with stabilization of the results after 6 months (medium term).

The acoustic results showed improvement presented in the different stages of evaluation (pre, short, medium and long term) (Table 2). The variables that displayed significant differences were PPQ and Shimmer, which were decreased if compared to baseline. On the acoustic analysis, there were significant differences when comparing overall improvement and partial improvement for Jitter (*p* = 0.016) and PPQ (*p* = 0.006) parameters (Table 3).

**Table 2:** Comparison of acoustic assessment data at the four time points.

Categories	PT	ST	MT	LT	*p*
	Data	Data	Data	Data	
*Fo – men*	(*n* = 28)	(*n* = 28)	(*n* = 14)	(*n* = 7)	
Min–max	84.7–204.7	84–177.5	84–127.2	84–127.2	
Median	115.3	127.2	96.5	94.392	
Mean ± SD	127.4 ± 43.4	128.5 ± 30.7	104.1 ± 18.657	100.3 ± 18.889	0.999

*Fo – women*					
Min–max	186–259.7	126.8–259.7	208.8–259.7	208.8–227.8	
Median	205.5	208.8	232.0	216.7	
Mean ± SD	197.9 ± 55.2	204.4 ± 36.937	232.8 ± 15.932	217.8 ± 9.546	0.999

*Jitter (0.633* *±* *0.351)*					
Min–max	1.218–10.443	1.197–6.355	1.203–3.662	2.266–3.662	
Median	2.718	2.056	2.067	2.964	
Mean ± SD	3.398 ± 2.254	2.686 ± 1.695	2.041 ± 0.861	2.964 ± 0.987	0.999

*PPQ (0.366* *±* *0.235)*					
Min–max	0.256–6.945	0.164–3.877	0.164–2.398	0.169–2.398	
Median	1.550	0.368	0.644	0.308	
Mean ± SD	1.963 ± 1.448	0.857 ± 0.961	0.733 ± 0.623	0.722 ± 0.841	0.010

*vFo (1.149* *±* *1.005)*					
Min–max	1.213–33.574	1.039–7.920	1.039–4.594	1.262–4.594	
Median	3.155	2.656	1.713	2.256	
Mean ± SD	4.613 ± 6.121	3.352 ± 2.491	2.168 ± 1.116	2.592 ± 1.576	0.147

Shimmer (1.997 ± 0.791)					
Min–max	1.787–32.859	1.210–11.201	1.378–8.977	1.378–8.977	
Median	5.855	3.060	3.046	2.690	
Mean ± SD	7.375 ± 5.868	4.072 ± 2.811	3.823 ± 3.046	4.271	0.001

*APQ (1.397* *±* *0.527)*					
Min–max	1.382–32.859	1.080–8.228	1.070–6.168	1.367–6.168	
Median	4.148	2.394	2.352	2.331	
Mean ± SD	5.332 ± 4.814	3.125 ± 2.030	2.769 ± 1.753	3.299 ± 2.284	0.151

*vAm (10.743* *±* *5.6)*					
Min–max	5.433–43.694	7.873–19.139	4.887–17.506	4.887–17.506	
Median	13.656	10.865	9.168	9.703	
Mean ± SD	16.548 ± 9.380	11.809 ± 3.328	10.473 ± 3.927	11.166 ± 4.985	0.998

*VTI (0.046* *±* *0.012)*					
Min–max	0.02–0.15	0.02–0.11	0.01–0.07	0.03–0.07	
Median	0.06	0.04	0.04	0.05	
Mean ± SD	0.06 ± 0.03	0.05 ± 0.02	0.04 ± 0.01	0.05 ± 0.01	0.114

SD, standard deviation; PT, pre-therapy; ST, short term; MT, medium term; LT, long term.

**Table 3:** Comparison of data between acoustic group evaluation of “overall improvement” and “partial improvement”.

Categories	Overall improvement (*n* = 16)	Partial improvement (*n* = 10)	*p*
	*n* (%)/data	*n* (%)/data	
*Fo – men (n* *=* *8)*	(*n* = 2)	(*n* = 5)	
Min–max	94–177	92 – 204	
Median	136	115	
Mean ± SD	136 ± 58.4	132 ± 48.8	0.857

*Fo – women (n* *=* *19)*	(*n* = 14)	(*n* = 5)	
Min–max	186–259	139–245	
Median	201	228	
Mean ± SD	192 ± 59.8	212 ± 41.8	0.257

*Jitter (0.633* *±* *0.351)*			
Min–max	1.218–5.532	1.231–10.443	
Median	2.326	4.196	
Mean ± SD	2.463 ± 1.156	5.031 ± 2.894	0.016

*PPQ (0.366* *±* *0.235)*			
Min–max	1.100–3.322	1.306–6.945	
Median	1.670	3.425	
Mean ± SD	1.741 ± 0.639	3.346 ± 1.675	0.006

*vFo (1.149* *±* *1.005)*			
Min–max	1.213–6.873	1.436–33.574	
Median	2.620	5.849	
Mean ± SD	2.695 ± 1.432	7.943 ± 9.902	0.027

*Shimmer (1.997* *±* *0.791)*			
Min–max	1.787–15.062	2.974–32.859	
Median	5.423	6.769	
Mean ± SD	6.158 ± 3.674	9.250 ± 8.612	0.182

*APQ (1.397* *±* *0.527)*			
Min–max	1.382–11.181	2.099–27.237	
Median	3.619	4.919	
Mean ± SD	4.321 ± 2.639	6.970 ± 7.288	0.121

*vAm (10.743* *±* *5.698)*			
Min–max	5.433–36.578	5.620–43.694	
Median	14.407	10.684	
Mean ± SD	15.739 ± 6.815	15.459 ± 12.296	0.363

*VTI (0.046* *±* *0.012)*			
Min–max	0.02–0.11	0.03–0.15	
Median	0.05	0.06	
Mean ± SD	0.05 ± 0.02	0.07 ± 0.03	0.135

SD, standard deviation.

Comparisons of variables related to VHI questionnaire over time also displayed significant differences for all variables. For the overall score, there was a difference pre therapy compared with other time points (values were, on average, higher than at other time points) (Table 4).

**Table 4:** Comparison of central tendency and variability measures related to different aspects (functional, organic, emotional) and total score of VHI between the Pre-Treatment (PT, *n* = 28), Short-Term (ST, *n* = 28), Medium-Term (MT, *n* = 14) and Long-Term evaluations (LT, *n* = 7).

Categories	PT	ST	MT	LT	*p*
	Data	Data	Data	Data	
*General*	(*n* = 28)	(*n* = 28)	(*n* = 14)	(*n* = 7)	
Min–max	3–97	0–79	0–29	0–29	
Median	54	18.5	12	9	
Mean ± SD	50.9 ± 22.4	16.9 ± 4.0	9.2 ± 8.7	9.9 ± 10.2	0.001

*Emotional*					
Min–max	0–32	0–23	0–5	0–3	
Median	12	0	0	2	
Mean ± SD	11.8 ± 8.3	3.6 ± 2.7	1.4 ± 1.7	1.4 ± 1.1	0.001

*Functional*					
Min–max	0–35	0–32	0–12	0–12	
Median	20	1	1	2	
Mean ± SD	18.7 ± 9.1	6.1 ± 4.9	3.6 ± 2.9	3.6 ± 4.5	0.001

*Organic*					
Min–max	2–39	0–24	0–14	0–14	
Median	23	2	4	4	
Mean ± SD	20.9 ± 8.3	6.1 ± 5.2	42 ± 3.9	5 ± 5	0.001

SD, standard deviation; PT, pre-therapy; ST, short term; MT, medium term; LT, long term.

Table 5 presents the results of the logistic regression used to determine the risk factors for non-improvement. The results indicated that Jitter entered the final model as a risk factor for partial improvement. For every unit of increased Jitter, there was an increase of 0.1% (1.001) of the patient chance for partial improvement, which indicates an increase in the no full improvement chance during rehabilitation.

**Table 5:** Jitter logistic regression correlation.

Categories	Descriptive level (*p*-value)	Odds ratio (OR)	Lower limit	Upper limit
Jitter	0.049	1.001	1.000	1.002
Constant	0.034	0.05		

## Discussion

Some studies demonstrate the effectiveness of voice therapy rehabilitation for UVFP.^19^, ^20^, ^21^, ^22^, ^23^, ^24^, ^25^, ^26^, ^27^, ^28^, ^29^, ^30^, ^31^, ^32^, ^33^, ^34^ The present study confirms the voice improvement for medium and long term rehabilitation.

The variables pitch, loudness and MPT also displayed improvement when comparing previous and immediately post therapy evaluation results; however, when comparing the results at the medium and long term evaluations, the differences were not significant. These data show that the results achieved during therapy were maintained over time, but without progressive improvement after 6 months, suggesting vocal quality stabilization.

Acoustic analysis results (PPQ and Shimmer) indicated improvement over time. These values were on average lower in the short, medium and long term when compared to the initial assessment.

In cases with continuing permanent vocal fold paralysis, glottal closure improvement became complete in most of the patients when compared with the initial otorhinolaryngology evaluation. The presented data is consistent with other studies that have evaluated the voice rehabilitation effectiveness. Different factors are involved in glottis closure. For example, interarytenoid musculature and cricothyroid muscle action can also help in the vocal fold medial movement and finally the inferior pharyngeal constrictor muscle, which can also help glottal closure. Based on the results found after voice therapy, we suggest that all of these settings can be favored by the techniques used during speech rehabilitation.

The consulted literature did not present data defining risk factors for unsatisfactory development in voice therapy in patients with unilateral vocal fold paralysis. The sample characteristics were similar in the groups with total and partial improvement. The auditory perceptual evaluation results, otorhinolaryngology evaluation and VHI had no significant differences.

In this study, the acoustic measures were the only factor directly related to UVFP patient improvement. The literature data suggest that voice disorder analysis, besides being an easy procedure, can indirectly and noninvasively measure laryngeal function and determine the vocal fold vibration condition.^35^

The Jitter and Shimmer parameters are widely used in scientific and clinical performance to predict diagnoses, as well as to document and evaluate dysphonia treatment.^36^, ^37^, ^38^ Different authors suggest that Jitter and Shimmer can be important predictors for changes on laryngeal physiology diagnosis.^39^, ^40^ However, there is controversy regarding Jitter and Shimmer analysis because of low reliability, sensitivity and specificity when voices with high roughness, low pitch and aperiodic signal are evaluated.^41^

In the study sample, we found the acoustic measures: Jitter, PPQ and VFO as associated factors for UVFP patient improvement. Those with partial improvement displayed, on average, higher values than these acoustic parameters. Moreover, Jitter measurement was considered a risk factor for the lack of total improvement.

Studies indicate that there is a fundamental frequency influence on Jitter results. Higher fundamental frequency values result in greater Jitter.^42^, ^43^ The fundamental frequency, Jitter and Shimmer influences are not yet fully understood, and there is a need to conduct new studies to determine the reasons for these relationships.^44^, ^45^

Considering the aforementioned aspects, we believe that the rate of patients with falsetto voice may have influenced the findings in this study, as this behavior can be found in patients with impaired vocal fold mobility. Brockmann et al.^46^ found that vocal intensity has a strong impact on Jitter and Shimmer measures. The fundamental frequency had relatively little influence.

Ortega et al.^40^ conducted a study on patients undergoing thyroid surgeries to determine whether subjective and acoustics voice evaluations could complement or replace laryngoscopy. Seventy-four patients were evaluated before and after surgery and submitted to acoustic analysis, subjective evaluation with GRBASI scale and nasofibrolaryngoscopy. The results indicated that Jitter, noise and harmonic variation proportion displayed the most frequent variations between the first and second evaluations (36% and 31%, respectively). Seven days after surgery, 5 (8%) patients were diagnosed with vocal fold paralysis, with a recovery of 2 cases after one month (5%). GRBASI values, Jitter and Shimmer displayed differences between patients with and without vocal fold paralysis (*p* < 0.05) and (*p* < 0.02). When there was a change in three voice parameters, the vocal fold paralysis was confirmed by laryngoscopy. The authors concluded that vocal fold paralysis might be observed in patients undergoing thyroidectomy, when there is a change in the GRBASI scale, Jitter or major changes of three acoustic analysis parameters. Furthermore, laryngoscopy should be performed only when these parameters have changed.

Observation of these parameters (fundamental frequency, Jitter and Shimmer) is well established as a noninvasive and objective method to quantitatively evaluate dysphonia degree and some different aspects of vocal fold paralysis. These parameters are used to describe normal and pathological voices while providing an objective method that can evaluate treatment clinical efficacy.^47^, ^48^

Changes in Jitter for UVFP patients should be evaluated more carefully because patients with major changes in this measure may have a worse voice rehabilitation prognosis.

## Conclusion

The results obtained in this study indicate that voice rehabilitation improves auditory perceptual, acoustic voice parameters and VHI parameters, in addition to favoring glottal closure in patients with vocal fold paralysis. The results were also stable over a one-year period. The Jitter value, when elevated, is a risk factor for voice therapy success.

## Conflicts of interest

The authors declare no conflicts of interest.
